# Strategies to Combat Multi-Drug Resistance in Tuberculosis

**DOI:** 10.1021/acs.accounts.0c00878

**Published:** 2021-04-22

**Authors:** Vinayak Singh, Kelly Chibale

**Affiliations:** † Drug Discovery and Development Centre (H3D), University of Cape Town, Rondebosch 7701, South Africa; ‡ South African Medical Research Council Drug Discovery and Development Research Unit, Department of Chemistry and Institute of Infectious Disease and Molecular Medicine, University of Cape Town, Rondebosch 7701, South Africa

## Abstract

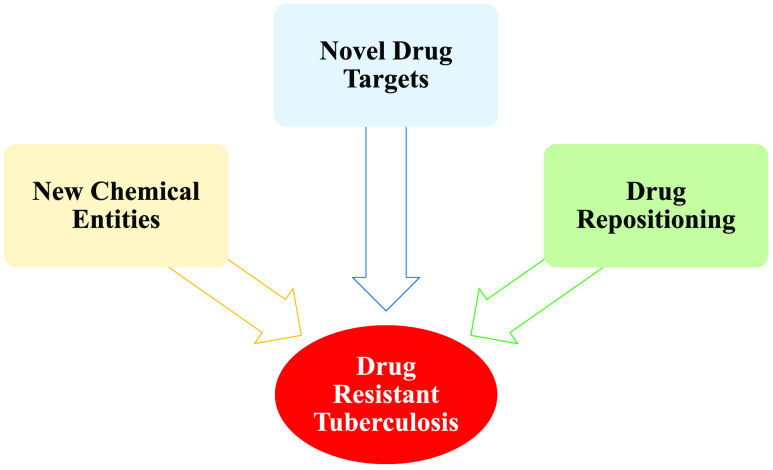

“*Drug resistance is an unavoidable consequence of the use of drugs; however, the emergence of multi-drug resistance can be managed by accurate diagnosis and tailor-made regimens.*”

Antimicrobial resistance (AMR), is one of the most paramount health perils that has emerged in the 21st century. The global increase in drug-resistant strains of various bacterial pathogens prompted the World Health Organization (WHO) to develop a priority list of AMR pathogens. *Mycobacterium tuberculosis* (*Mtb*), an acid-fast bacillus that causes tuberculosis (TB), merits being one of the highest priority pathogens on this list since drug-resistant TB (DR-TB) accounts for ∼29% of deaths attributable to AMR. In recent years, funded collaborative efforts of researchers from academia, not-for-profit virtual R&D organizations and industry have resulted in the continuous growth of the TB drug discovery and development pipeline. This has so far led to the accelerated regulatory approval of bedaquiline and delamanid for the treatment of DR-TB. However, despite the availability of drug regimes, the current cure rate for multi-drug-resistant TB (MDR-TB) and extensively drug-resistant TB (XDR-TB) treatment regimens is 50% and 30%, respectively. It is to be noted that these regimens are administered over a long duration and have a serious side effect profile. Coupled with poor patient adherence, this has led to further acquisition of drug resistance and treatment failure. There is therefore an urgent need to develop new TB drugs with novel mechanism of actions (MoAs) and associated regimens.

This Account recapitulates drug resistance in TB, existing challenges in addressing DR-TB, new drugs and regimens in development, and potential ways to treat DR-TB. We highlight our research aimed at identifying novel small molecule leads and associated targets against TB toward contributing to the global TB drug discovery and development pipeline. Our work mainly involves screening of various small molecule chemical libraries in phenotypic whole-cell based assays to identify hits for medicinal chemistry optimization, with attendant deconvolution of the MoA. We discuss the identification of small molecule chemotypes active against *Mtb* and subsequent structure–activity relationships (SAR) and MoA deconvolution studies. This is followed by a discussion on a chemical series identified by whole-cell cross-screening against *Mtb*, for which MoA deconvolution studies revealed a pathway that explained the lack of in vivo efficacy in a mouse model of TB and reiterated the importance of selecting an appropriate growth medium during phenotypic screening. We also discuss our efforts on drug repositioning toward addressing DR-TB. In the concluding section, we preview some promising future directions and the challenges inherent in advancing the drug pipeline to address DR-TB.

## Key References

- van der WesthuyzenR.; WinksS.; WilsonC. R.; BoyleG. A.; GessnerR. K.; Soares de MeloC.; TaylorD.; de KockC.; NjorogeM.; BrunschwigC.; LawrenceN.; RaoS. P.; SirgelF.; van HeldenP.; SeldonR.; MoosaA.; WarnerD. F.; AristaL.; ManjunathaU. H.; SmithP. W.; StreetL. J.; ChibaleK.Pyrrolo[3,4-c]pyridine-1,3(2H)-diones: A Novel Antimycobacterial Class Targeting Mycobacterial Respiration. J. Med. Chem.2015, 58, 9371–93812655124810.1021/acs.jmedchem.5b01542.^1^*This work identified a novel class of inhibitors that bind to the QcrB subunit of cytochrome bc1 in Mycobacterium tuberculosis.*
- WilsonC. R.; GessnerR. K.; MoosaA.; SeldonR.; WarnerD. F.; MizrahiV.; Soares de MeloC.; SimelaneS. B.; NchindaA.; AbayE.; TaylorD.; NjorogeM.; BrunschwigC.; LawrenceN.; BoshoffH. I. M.; BarryC. E.; SirgelF. A.; van HeldenP.; HarrisC. J.; GordonR.; Ghidelli-DisseS.; PflaumerH.; BoescheM.; DrewesG.; SanzO.; SantosG.; Rebollo-LopezM. J.; UronesB.; SelenskiC.; Lafuente-MonasterioM. J.; AxtmanM.; LelievreJ.; BallellL.; MuellerR.; StreetL. J.; GhorpadeS. R.; ChibaleK.Novel Antitubercular 6-Dialkylaminopyrimidine Carboxamides from Phenotypic Whole-Cell High Throughput Screening of a SoftFocus Library: Structure-Activity Relationship and Target Identification Studies. J. Med. Chem.2017, 60, 10118–101342914875510.1021/acs.jmedchem.7b01347PMC5748279.^2^*A novel scaffold with a unique mode of action.*
- Soares de MeloC.; SinghV.; MyrickA.; SimelaneS. B.; TaylorD.; BrunschwigC.; LawrenceN.; SchnappingerD.; EngelhartC. A.; KumarA.; ParishT.; SuQ.; MyersT. G.; BoshoffH. I. M.; BarryC. E.; SirgelF. A.; van HeldenP. D.; BuchananK. I.; BaylissT.; GreenS. R.; RayP. C.; WyattP. G.; BasarabG. S.; EyermannC. J.; ChibaleK.; GhorpadeS. R.Antitubercular 2-Pyrazolylpyrimidinones: Structure-Activity Relationship and Mode-of-Action Studies. J. Med. Chem.2021, 64, 719–7403339528710.1021/acs.jmedchem.0c01727PMC7816196.^3^*A novel scaffold perturbing Fe-homeostasis of Mycobacterium tuberculosis.*
- AkesterJ. N.; NjariaP.; NchindaA.; Le ManachC.; MyrickA.; SinghV.; LawrenceN.; NjorogeM.; TaylorD.; MoosaA.; SmithA. J.; BrooksE. J.; LenaertsA. J.; RobertsonG. T.; IoergerT. R.; MuellerR.; ChibaleK.Synthesis, Structure–Activity Relationship, and Mechanistic Studies of Aminoquinazolinones Displaying Antimycobacterial ActivityACS Infect. Dis.2020, 6, 1951–19643247028610.1021/acsinfecdis.0c00252PMC7359024.^4^*This work highlights the importance of performing secondary screening in multiple growth media.*

## 1 Introduction

**Figure 1:**
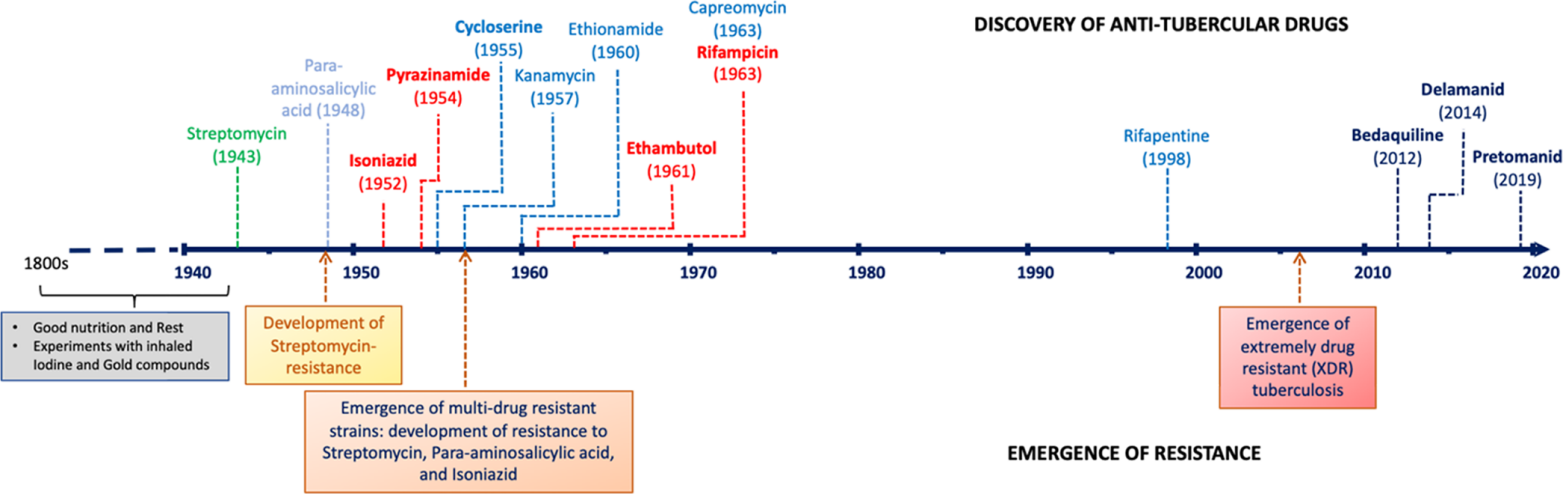
Timeline of TB drug discovery and emergence of resistance. Dates are associated with the publication/approval.

Tuberculosis (TB), caused by the bacillus *Mycobacterium tuberculosis* (*Mtb*), is a chronic necrotizing infection, which shows a wide variety of manifestations. Historically, TB was considered incurable until the discovery and use of streptomycin (STR) in 1946 (Figure 1). In the very first clinical trial conducted by the British Medical Research Council (BMRC), STR showed an impressive reduction in mortality but very soon resistance emerged to this drug.^5^ In the 1960s, a trial of isoniazid (INH)-para-aminosalicylic acid (PAS) was launched, which suggested that care in the home was equally effective and comparable to treatment in a sanatorium or hospital.^6^

Modern-day standard TB chemotherapy effective at treating drug-susceptible (DS) disease requires 6 months of administration using a combination regimen containing INH, rifampicin (RIF), pyrazinamide (PZA), and ethambutol (EMB). The emergence of HIV/TB comorbidity led to the declaration of TB as a global public health emergency by the World Health Organization (WHO) in 1993. This is further worsened by a rise in diabetes which results in a more than 3-fold increase in the risk of TB, a bigger risk than HIV in certain regions.^7^ However, the most significant factor affecting this global health menace is the emergence of drug resistance where the treatment runs up to 2 years in some cases with limited success and is accompanied by severe toxicity. In this Account, we evaluate the challenges facing TB drug discovery and associated drug resistance, focusing on key insights and highlighting the outstanding questions. We also discuss how these might be tackled. We conclude with a perspective on potential areas of future research in TB drug discovery focusing on circumventing drug resistance.

## 2 Drug Resistant TB: A Challenge

Contrasting with other bacterial pathogens that have evolved to spread drug resistance in populations via horizontal gene transfer, drug resistance in *Mtb* is mainly due to mutations in chromosomal genes (Table 1). This genotypic resistance may develop due to single nucleotide polymorphisms (SNPs) and insertions or deletions in bacterial genes, affecting prodrug activation, modifications in the drug-target structure, reduced drug permeability, or increased efflux.^8−10^ However, and interestingly, the development of resistance in *Mtb* is complex. It involves an interplay of clinical, biological, and microbiological processes, e.g., nonadherence of patients to the therapy^11^ leading to the development of genetic resistance, complexity of granulomas which presents a barrier to effective drug distribution^12^ and thus limiting adequate supply of drugs to *Mtb*, intrinsic resistance, phenotypic resistance exhibited by nonreplicating (NR) drug-tolerant bacteria,^13^ and acquired resistance. Drug-resistance in TB can be categorized in four categories: (i) Single drug-resistant TB (SDR-TB), where only one drug of the combination therapy is subject to resistance; (ii) multi-drug-resistant TB (MDR-TB), defined as the failure to respond to at least RIF and INH; (iii) extensively drug-resistant TB (XDR-TB), when MDR-TB coupled with the resistance to at least one of the second-line drugs; and (iv) totally drug-resistant (TDR-TB), when all the available first- and second-line anti-TB drugs are ineffective. At this juncture, it is noteworthy that, due to the limitations in drug-susceptibility testing of second-line drugs for resistance, the WHO has yet to recognize TDR-TB.

**Table 1:**
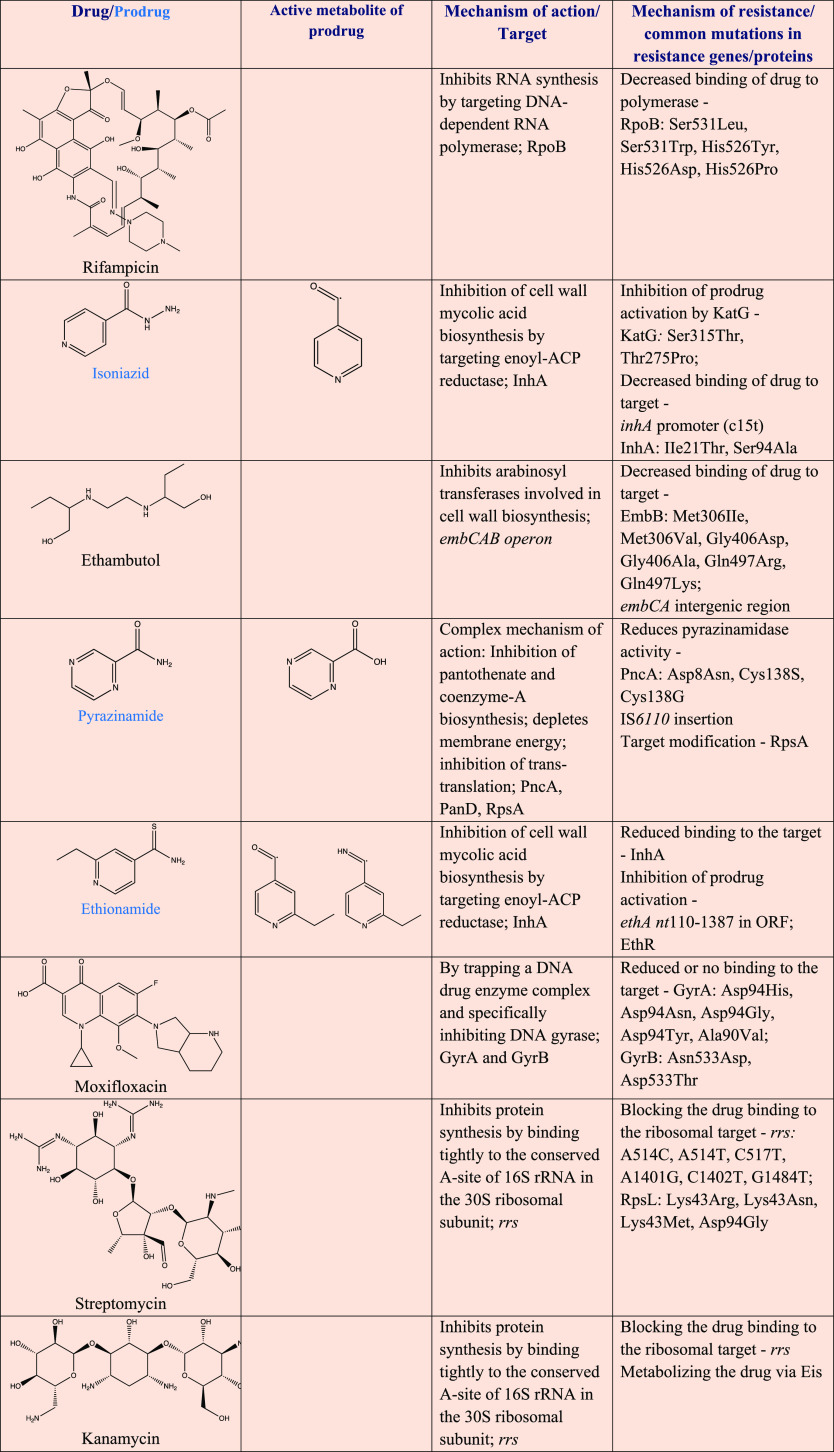

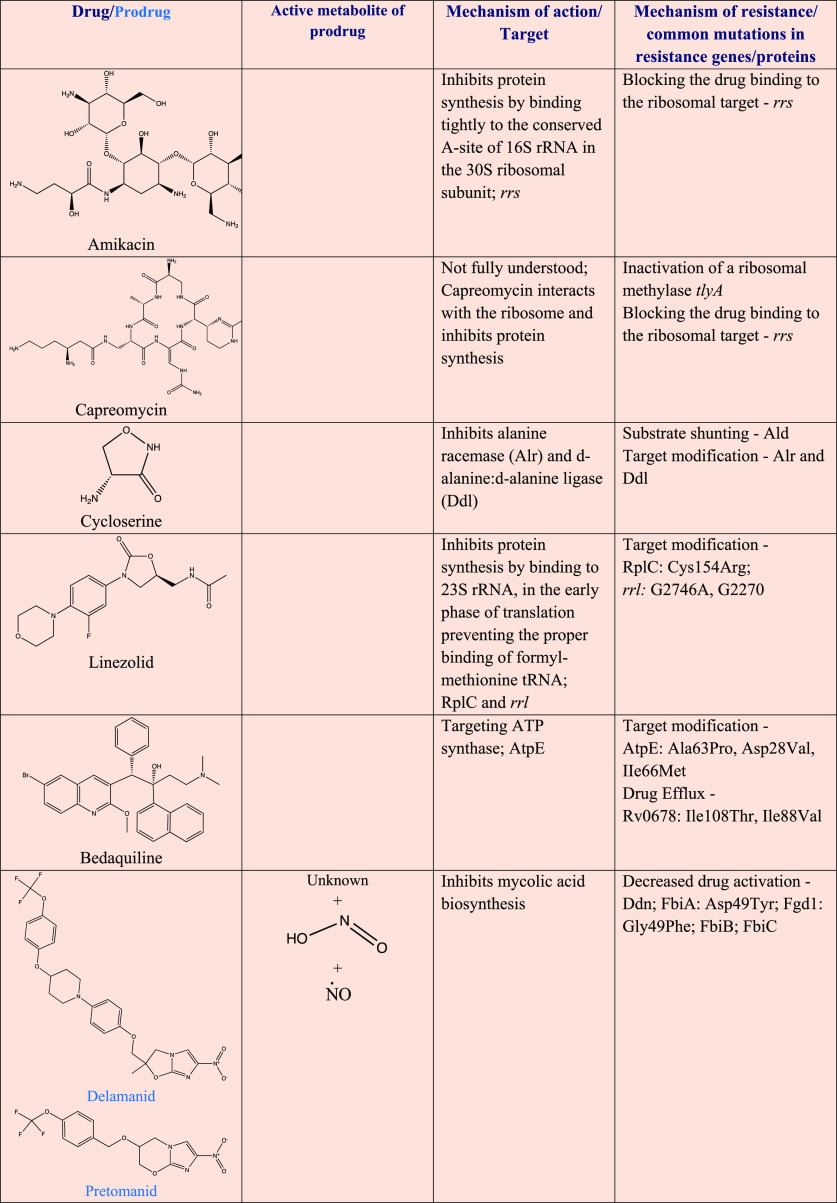
Drug Resistance Mechanisms of Common Anti-TB drugs

In 2019, globally an estimated 10 million people fell ill with TB. There were ∼1.2 million deaths recorded among HIV-negative and an additional 208 000 deaths of HIV positive people due to TB in 2019.^14^ Although TB affects people of both sexes, in 2019 men showed the highest-burden and accounted for 56%, women for 32%, and children (aged <15 years) for 12% of all TB cases. DR-TB continues to be a major threat. There were about 0.5 million new cases of rifampicin-resistant TB (RR-TB), and ∼78% of these had MDR-TB. Globally, there were 3.3% of new TB cases and 17.7% of previously treated cases that displayed MDR/RR-TB. A total of 12 350 globally reported cases of XDR-TB echo the mayhem of drug resistance in TB. The WHO has mandated *Mtb* drug susceptibility testing (DST) for all TB patients to better guide treatment decisions and improve treatment outcomes. However, the availability of resources in high burden countries remains a major problem. Traditionally, phenotypic drug susceptibility testing is the first choice for the diagnosis of MDR/XDR-TB, but it can take ∼12 weeks to deliver results which directly affects effective treatment due to the delay in initiating therapy. Toward this, a single diagnostic test involving all possible mutations in the resistance genes’ profile would be the ultimate assay. And this fast and accurate diagnosis should subsequently result in effective treatment and reduction in resistance development. However, DR-TB may also spread when a new infection occurs with a resistant *Mtb* strain, underscoring the prevention of treatment failure to stop the spread of MDR/XDR infections.^15−17^ For the effective treatment of DR-TB, the WHO has issued recommendations with regard to drug combinations to be used, length of treatment, and how patient response should be monitored.^18^ The MDR/RR-TB regimens can be offered as either (i) a standardized shorter regimen of 9–12 months or (ii) longer regimens of up to 20 months.

## 3 Drugs and Regimens in Development

**Figure 2:**
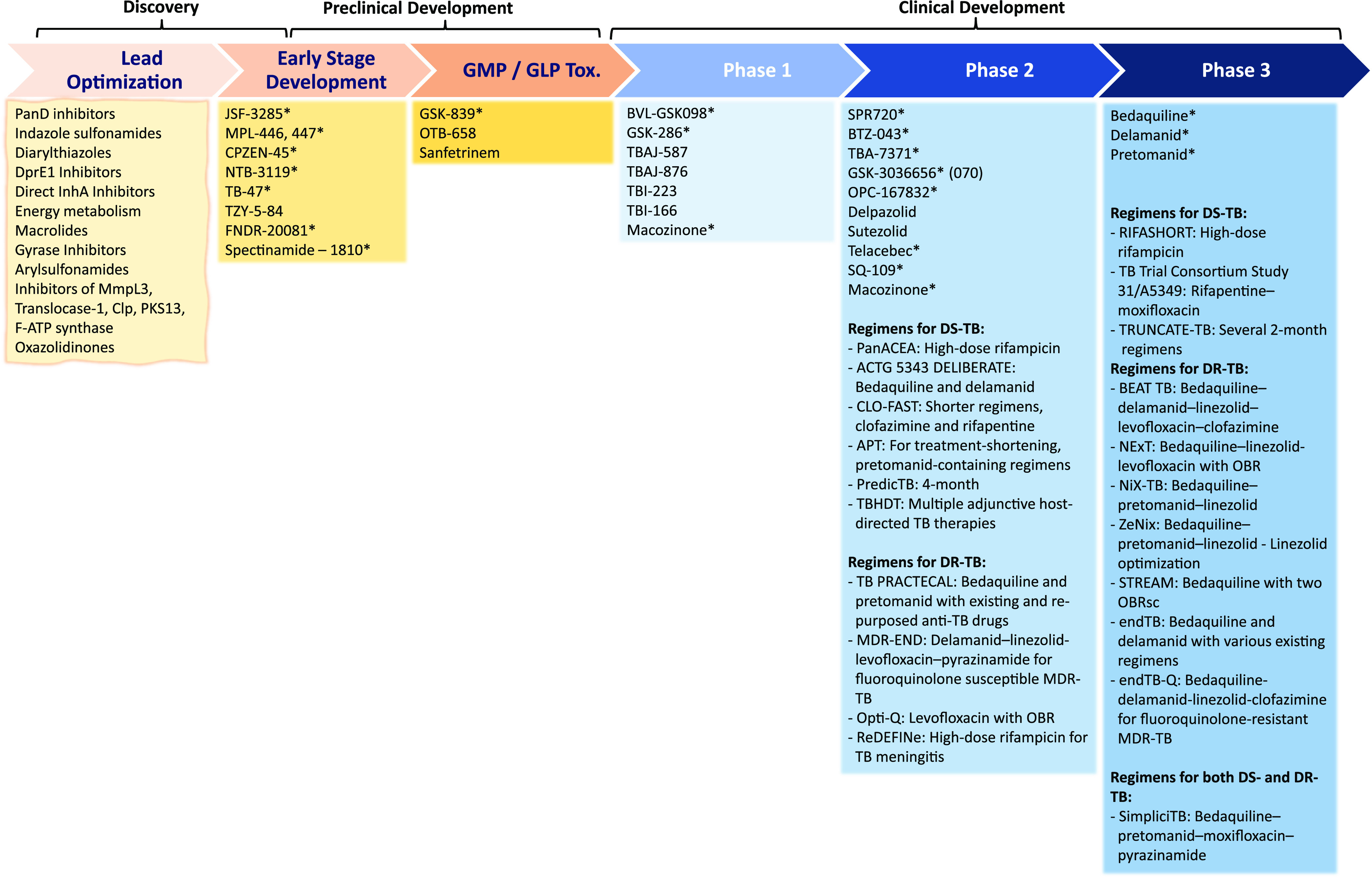
Current clinical development pipeline for anti-TB drugs and regimens, March 2021. *, new chemical class; OBR, optimized background regimen. (Adapted with permission from the Stop TB Partnership Working Group on New Drugs pipeline; for detailed information, please see: http://www.newtbdrugs.org.)

Adequate treatment of DR-TB can prevent further development of new DR strains, which can worsen the current situation of poor prognosis and limited therapeutic options. Current second- and third-line anti-TB drugs are more toxic, less efficacious, and more expensive than first-line drugs. In light of this, the most important step toward the management of DR-TB is the use of an efficacious drug regimen, derived directly from the drug-susceptibility testing outcomes. This testing needs to be rapid in order to identify DR-TB which can allow for an immediate prescription of a specific drug regimen. Two recently approved anti-TB drugs, bedaquiline and delamanid, are used in many countries to treat MDR-TB when no other option is available. However, due to severe side effects and emergence of resistance to these, there is an urgent need for novel drugs and regimens.^19^ Regimens involving new drugs would be a dual feat toward achieving DR-TB treatment, as these would reduce the current requirement of drug-susceptibility testing. It is interesting to note that global efforts for TB treatment have dramatically evolved in the recent past (Table 2). A number of new anti-TB agents and repurposed drugs are under clinical development as shown in the pipeline (Figure 2).^14,20^

**Table 2:**
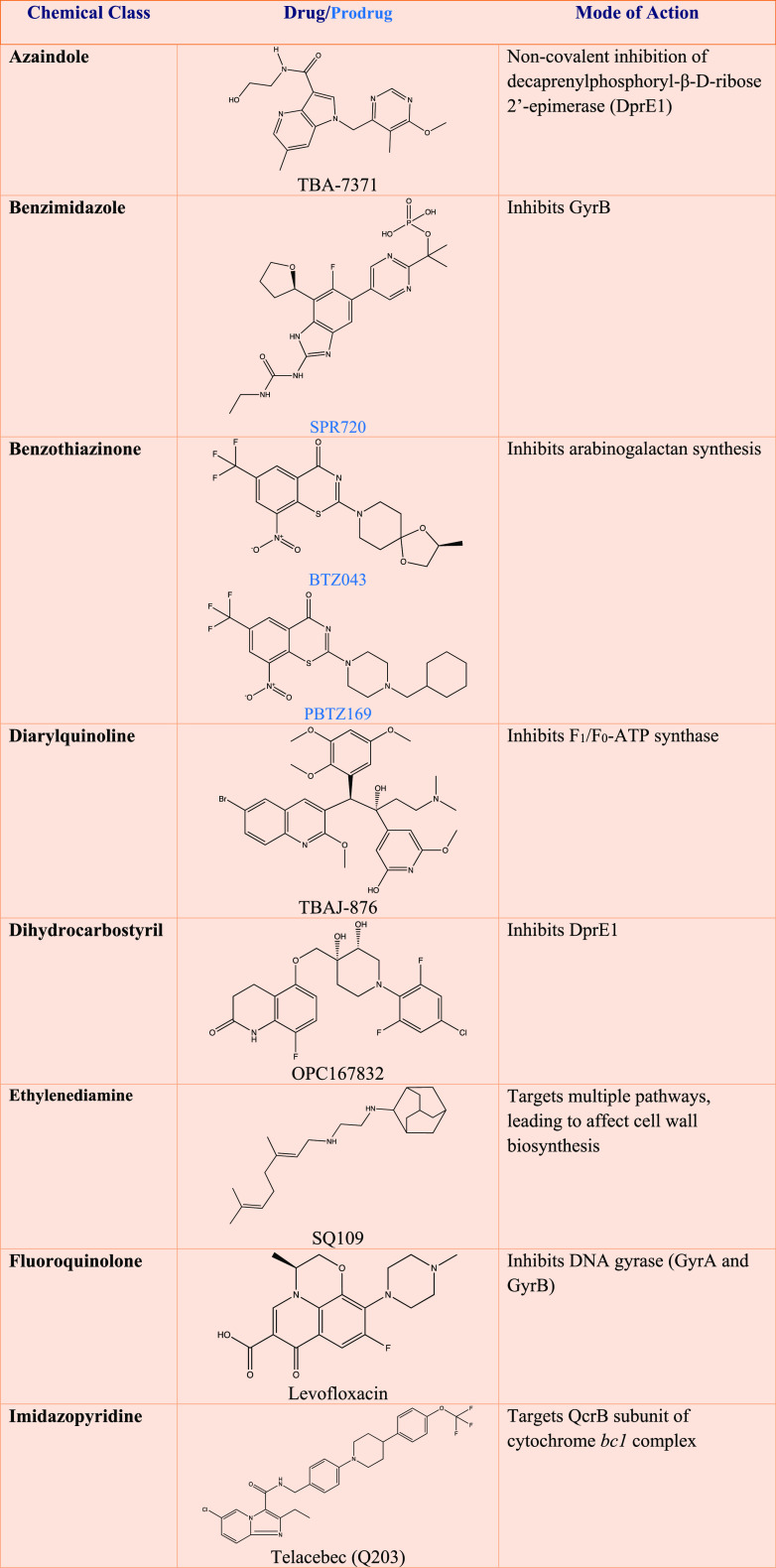

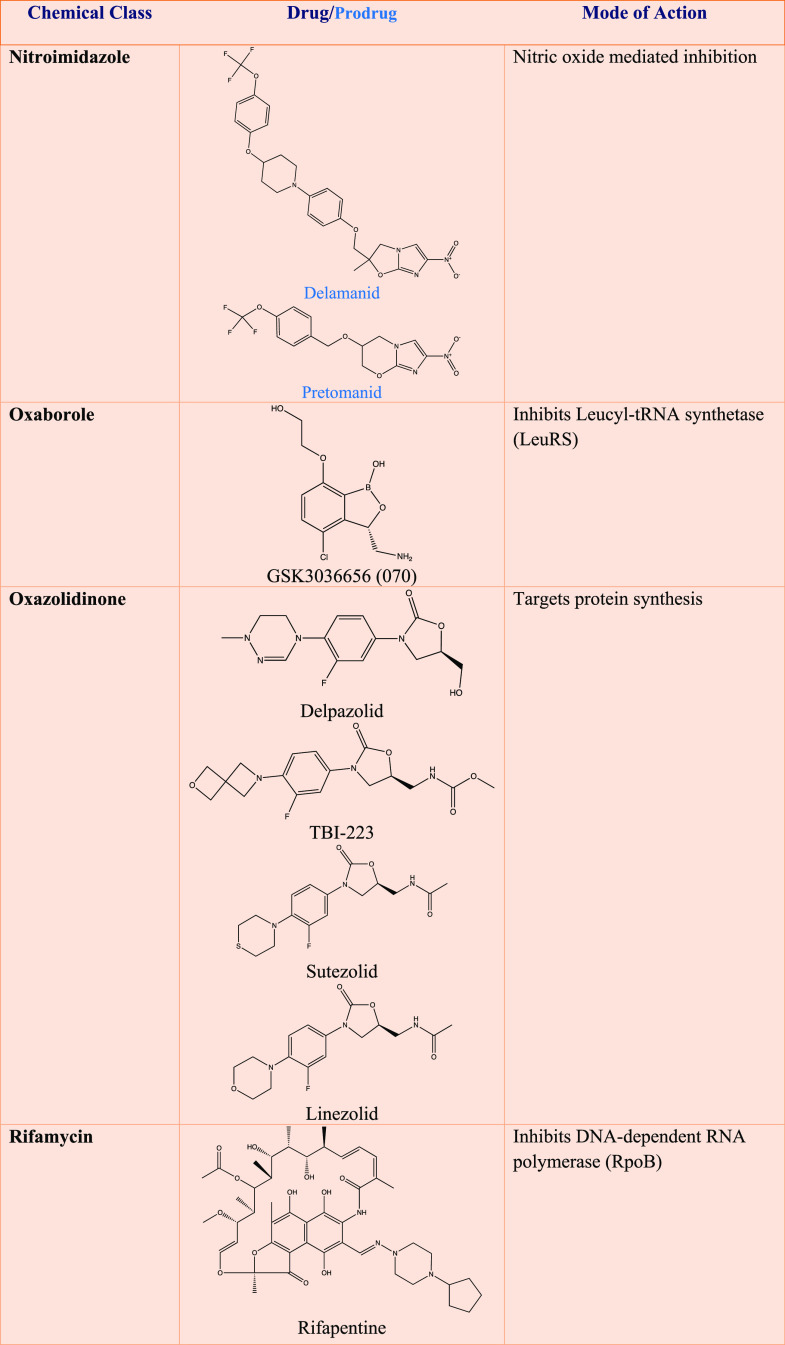

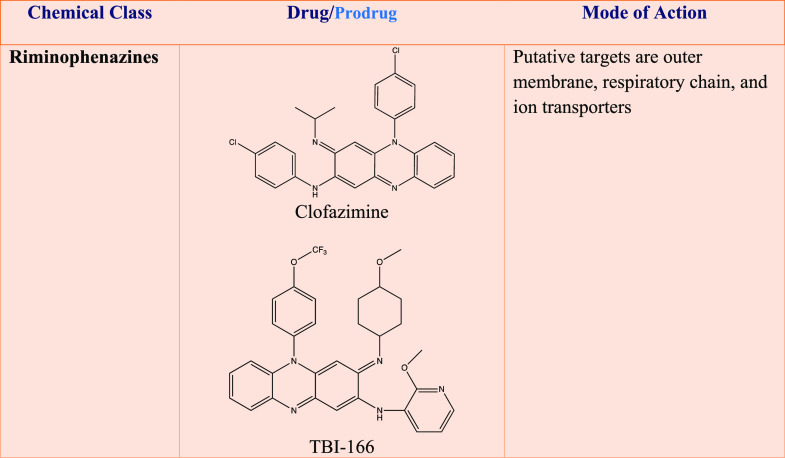
Promising Anti-tubercular Chemical Classes under Clinical Development to Address Drug Resistance^a^

a Detailed information can be found at: http://www.newtbdrugs.org.

## 4 DR-TB: How Can We Intervene?

**Figure 3:**
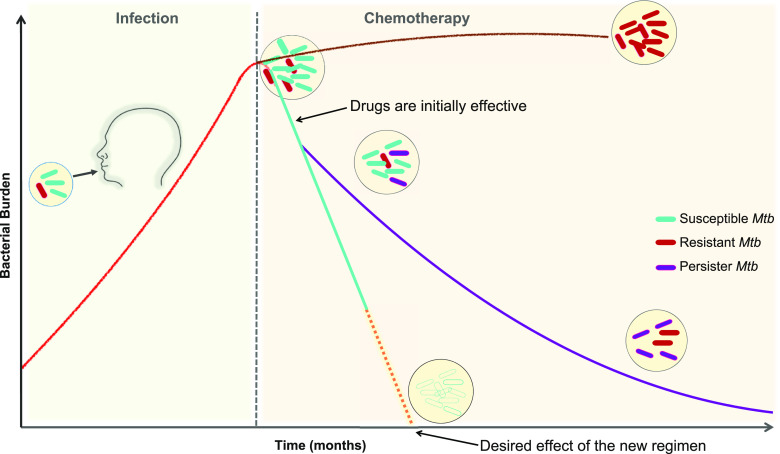
Kinetics of tuberculosis treatment. Here we consider a heterogeneous population of naturally occurring *M. tuberculosis* (*Mtb*) as an infectious dose. After the start of chemotherapy, there can be three possible outcomes: eradication of the disease (desired outcome), partially effective longer treatment due to the emergence of persister bacteria, and failure of the treatment due to the emergence of predominantly resistant *Mtb*. The presence of persister bacterial populations can be exemplified by a classic biphasic kill curve after the start of treatment: a brief period of rapid killing followed by a delayed killing.

As highlighted in Figure 1 and Table 1, resistance to newly discovered drugs has generally developed within 10 years of their first use. Genetic resistance to the drug is an evolutionary process in response to the selection pressure of the drug(s) that reinforces the need to continue optimizing new drug(s) and associated regimens. New vulnerable drug targets with at least three levels of validation, i.e., genetic, phenotypic, and in vivo, can be a basis for confronting DR-TB. In parallel, considering the host environment, identifying compounds targeting *Mtb* residing in granulomas/cavities is of interest. In addition, a micromanaged antibiotic stewardship strategy can be effective in slowing down the emergence of resistance. However, understanding antibiotic resistance in *Mtb* is not straightforward (Figure 3). Resistance can appear as a result of a persistent phenotype, which is displayed by drug-tolerant populations of *Mtb* known as persisters. These appear to be nonreplicating or slowly growing due to reduced metabolic activity and carry noninheritable phenotypic resistance. Multiple pathways can be involved in evolving persisters, such as energy metabolism, regulators, toxin-antitoxin system, and transporter or efflux mechanisms. Therefore, an understanding of persistence/dormancy/tolerance in *Mtb* may direct the rational development of new treatment regimens.^21^ Management of this tolerant/persister population would significantly slow down the development of drug resistance which will help in TB treatment shortening. However, it remains a challenge because drug-tolerant populations are generally tolerant of all drugs. Recent advances have seen the establishment of various in vitro models representing the nonreplicating or tolerant state. These models include low pH, hypoxia, nutrient starvation, carbon starvation, and a recently developed rapid, low pH nutrient stress to facilitate understanding of the physiology of the tolerant population and a quick determination of the bactericidal activity of test compounds.^22^ Also, induction of alternative pathways of prodrug activation can be a viable mechanism for neutralizing drug resistance, as exemplified by the SMARt-420 (Small Molecules Aborting Resistance) that stimulates ethionamide activity and reverses EthA-mediated resistance.^23^

## 5 Addressing Drug Resistant TB: Our Approach

**Figure 4:**
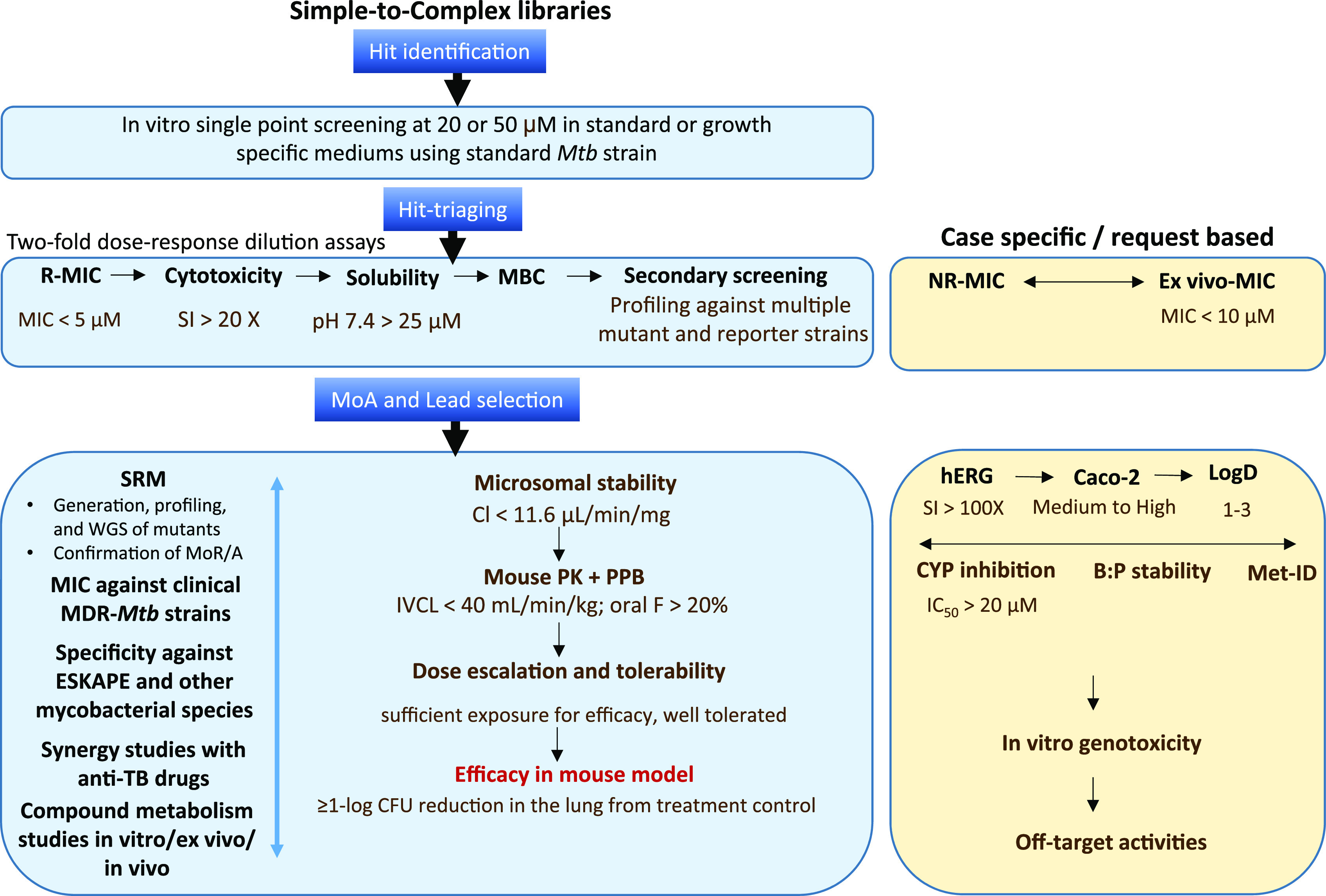
Early drug discovery test-cascade used at the Drug Discovery and Development Centre (H3D). *Mtb*, *M. tuberculosis* H37Rv; R, replicating; MIC, minimum inhibitory concentration (7–14 days); cytotoxicity, against VERO (kidney epithelial cells extracted from an African green monkey) and/or CHO (Chinese hamster ovarian) and/or HepG2 (human liver cancer) mammalian cell-lines; solubility, in fasted-state intestinal fluid (FaSSIF) kinetic-solubility assay at pH 6.5 and in PBS at pH 7.4; MBC, minimum bactericidal concentration (7–28 days); NR, nonreplicating, nutrient starvation/hypoxia/4-stress model; ex vivo, using RAW264.7, J774, and/or THP.1 derived macrophages; MoA, mechanism of action; SRM, spontaneous resistant mutant; WGS, whole-genome sequencing; PK, pharmacokinetics; PPB, plasma protein binding; F, bioavailability; CYP, Cytochrome P450; B:P, blood:plasma ratio; MetID, metabolite identification; MoR, mechanism of resistance; SI, selectivity-index; CL, clearance; IVCL, in vivo clearance; genotoxicity, AMES unless the compounds are active against Salmonella, mouse lymphoma, and mouse micronucleation assays; off-target activities, secondary pharmaceutical panels of enzymes, GPCRs, ion channels, receptors, transporters, etc. Dotmatics is used in data management.

As described above, most of the current drugs in clinical development for TB are derivatives of existing drug classes. To identify new chemical starting points for drug discovery, the target-based screening approach is preferred when target validation has been demonstrated. Selecting the target mainly relies on its vulnerability and selectivity. Interestingly, despite tremendous success in other disease areas, this approach was largely not successful in TB drug discovery due to compound permeability issues across the lipid-rich cell-wall, efflux, and metabolization of the compound in *Mtb*. On the other hand, the phenotypic whole-cell screening approach has relatively been more successful in delivering cell-permeable small molecules active against whole *Mtb* cells as starting points for TB drug discovery. This is then typically followed up by deconvolution of the MoA of the actives. Our program has largely utilized this approach with small molecule active hits being progressed through the drug discovery process, underpinned by medicinal chemistry optimization, toward improving potency, pharmacokinetic, and pharmacodynamic properties. A critical benchmark in selecting a “New Chemical Entity” from the screening campaign is the representation of a new drug class, acting via a novel MoA. To achieve this, we typically perform hit-triaging at an early stage to avoid rediscovery of the established targets by using a slew of secondary assays. The blueprint of our early drug discovery approach is summarized in the test cascade (Figure 4). Our whole-cell based phenotypic screening assesses the growth kinetics by measuring the minimum inhibitory concentration (MIC) as the end point. As the libraries of small polar molecules offer a significant edge by occupying a unique chemical space of MW (<250 Da) and lipophilicity (clogP < 2.5) in TB drug discovery, we screened such and several other libraries. Moreover, we have learned the importance of target deconvolution studies in order to drive successful structure–activity relationship (SAR) exploration. For this reason, we typically initiate target identification work in parallel with medicinal chemistry hit optimization studies.

**Figure 5:**
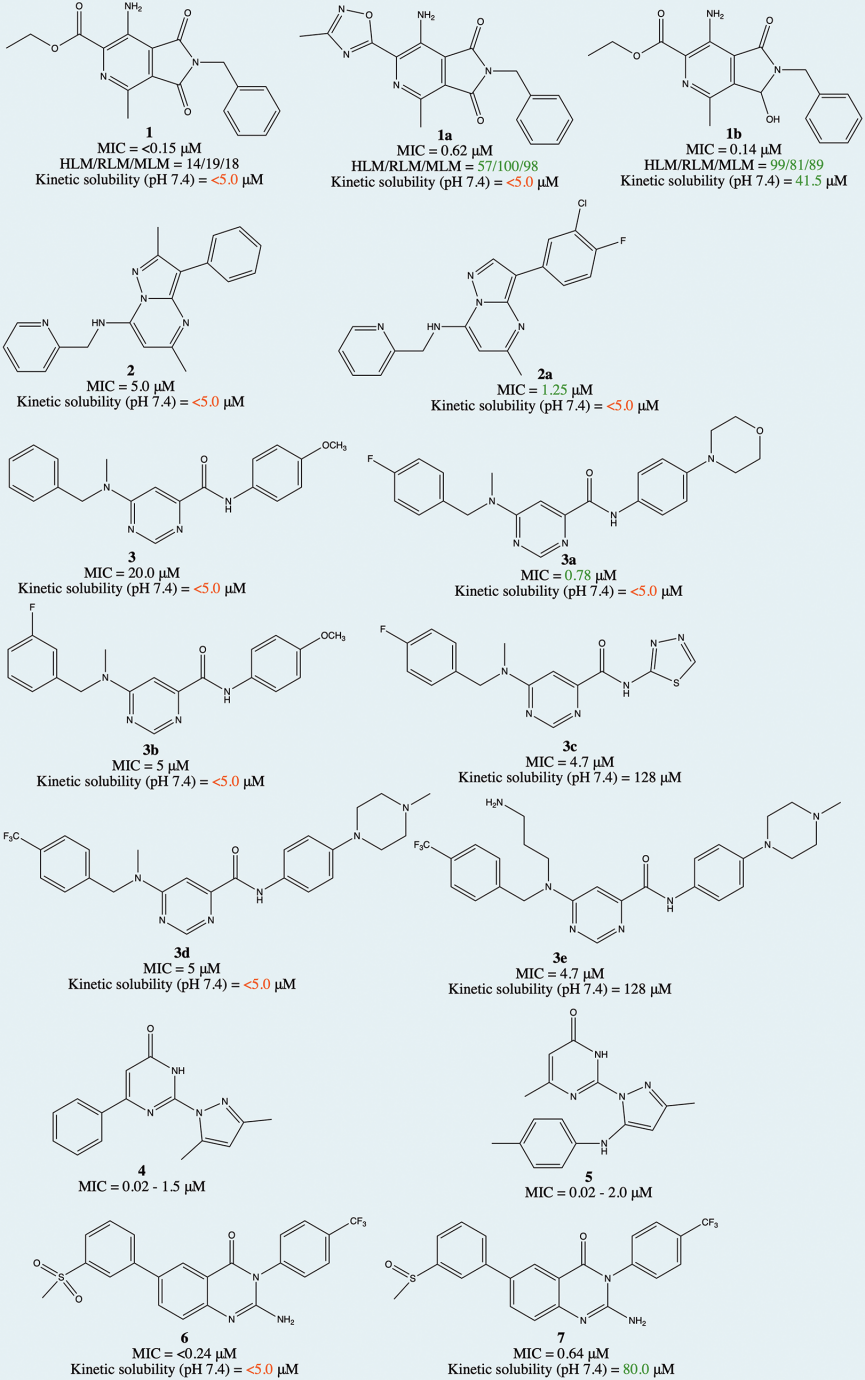
Structures of selected compounds explored for SAR and/or MoA.

**Figure 6:**
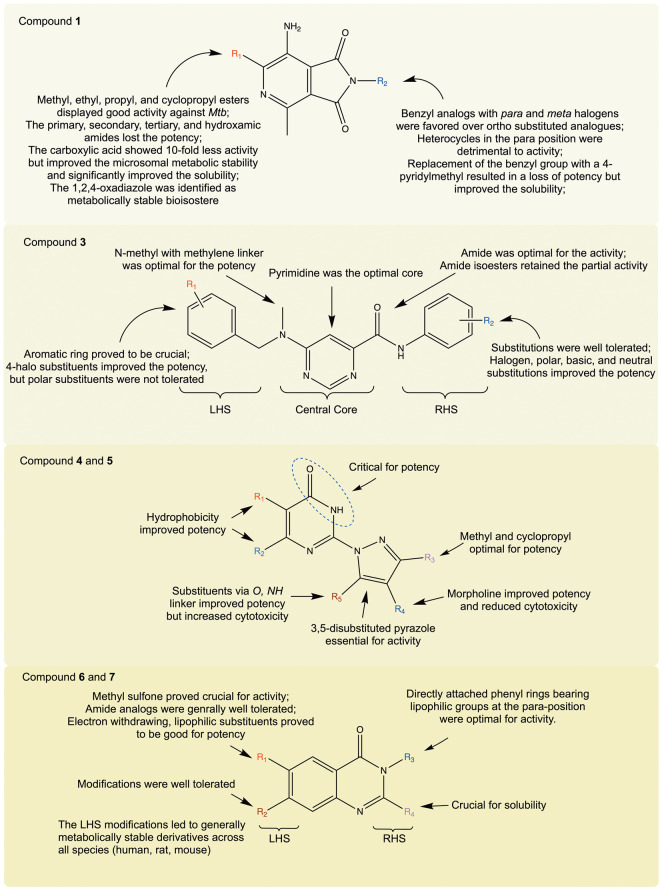
SAR summary of compounds **1**, **3**, **4**, **5**, **6**, and **7**. *Mtb*, *Mycobacterium tuberculosis*; LHS, left-hand side; RHS, right-hand side.

In this context, whole-cell screening of a small polar library of ∼6000 compounds in a novel chemical space (Mw 150–350 Da, clogP −1 to 3.5; assembled by Novartis Institute for Tropical Diseases (NITD)) identified a potent hit compound, the pyrrolo[3,4-*c*]pyridine-1,3(2H)-dione **1** (Figures 5 and 6).^1^ Subsequent resynthesis, retesting, and ADME profiling confirmed the good in vitro potency (<0.15 μM) against *Mtb*. However, the ester functionality raised concerns about its metabolic instability as less than 20% of the compound remained after 30 min of incubation with human, rat, and mouse liver microsomes. Therefore, the initial medicinal chemistry strategy was aimed at improving the metabolic liability of compound **1** while maintaining good anti-*Mtb* activity by identifying bioisosteric replacements for the labile ester. The SAR produced 1,2,4-oxadiazole **1a** with improved metabolic stability (HLM/RLM/MLM; % remaining, 57/100/98) over ester **1** (HLM/RLM/MLM; % remaining, 14/19/18) and significant anti-*Mtb* activity (0.62 μM). Additional SAR exploration delivered compound **1b** with good in vitro metabolic stability and excellent potency of the hit compound **1**. Next, in order to investigate the MoA of pyrrolo[3,4-*c*]pyridine-1,3(2*H*)-diones, we explored a chemo-genetic approach by attempting to raise spontaneous-resistant mutants (SRMs) in *Mtb*. SRM generation attempts were unsuccessful even when the bacterial cultures were exposed to compounds at a concentration of 2–200× MIC. Nonetheless, the hit-triaging of the series showed that the compounds are hyperactive to a nonessential cytochrome *bd* oxidase deletion mutant (Δ*cyd*KO). This led us to screen the compounds against a QcrB (Ala317Thr) mutant of *Mtb*; QcrB is an essential subunit of respiratory cytochrome *bc*_*1*_ complex and the target of telacebec (Q203), that has completed phase-2 clinical trials with promising results (ClinicalTrials.gov: NCT03563599).^24,25^ Compounds in the series showed resistance to the QcrB point mutant; therefore, in conjunction with the hyperactivity to the Δ*cyd*KO, this strongly suggested that the pyrrolo[3,4-*c*]pyridine-1,3(2*H*)-diones target the QcrB subunit of the cytochrome *bc_1_* complex. Next, to determine the clinical potential of this series, we evaluated the activity of this series against DS *Mtb* clinical isolates belonging to different lineages such as atypical Beijing, Haarlem, Cas 1/Delhi, and X families. The tested compounds were equally effective against all the strains with an MIC_90_ of 0.08–0.6 μM. Although, we could not test this series against DR clinical isolates it is to be noted that QcrB inhibitors are equally effective against DR isolates.^24,26^

A SoftFocus library^27^ comprising 35 000 compounds represented by >200 scaffolds that had primarily been designed to target specific gene families, including kinases, G-protein coupled receptors, and ion channels, was acquired. High-throughput screening against *Mtb* was carried out in Middlebrook 7H9 medium under aerobic conditions and glucose as a carbon source. Two hit series, one based on aminopyrazolo[1,5-*a*]pyrimidines and the other based on 6-dialkylaminopyrimidine carboxamides, were selected based on the potency, intellectual property overlap, and chemical-structural properties. As described above, the biology triage process facilitated the exclusion of compounds acting on promiscuous targets such as QcrB, MmpL3, and DprE1 as well as DNA damaging agents. In the aminopyrazolo[1,5-*a*]pyrimidine series,^28^ our medicinal chemistry efforts were focused on substitution with a variety of amino groups in the 7-position and aryl groups in the 3-position (compound **2**, Figure 5). This yielded compound **2a** with an MIC_99_ of 1.25 μM (vs original hit of 5 μM). However, this series exhibited liabilities of poor aqueous solubility and in vitro cytotoxicity. The series did not show MIC modulation against the tested mutant and reporter strains which is suggestive of the involvement of a novel MoA. In the 6-dialkylaminopyrimidine carboxamide series,^2^ compound **3** was identified as a suitable hit with a moderate potency of 20 μM (MIC), low toxicity (VERO cell-line, IC_50_ of 287 μM), but poor kinetic solubility (<5 μM) linked to the high lipophilicity and flat aromatic character of the molecule. To improve the properties, a detailed SAR focusing on understanding key hydrogen bond donor–acceptor interactions critical for activity, shape, and size of the central core and scope for substitution/modification on either side of the molecule was used (Figures 5 and 6). Additionally, the possibility of the addition of heteroatoms or polar groups was also investigated to reduce the lipophilicity, poor physicochemical properties, and structure alerts, e.g., presence of potentially AMES positive anilines upon cleavage of the amide group. This effort resulted in potency improvement (compound **3a**, 0.78 μM from 20 μM) but not solubility. Nonetheless, the detailed SAR demonstrated limitations as well as scope for further optimization of the series. The biology hit-triaging suggested involvement of a novel MoA. In order to supplement our MoA identification efforts, we raised a SRM with a distinct resistance phenotype against compound **3b**. The **3b**-resistant mutant did not exhibit cross-resistance to the standard TB drugs, suggesting a potential novel MoA for the series. Unfortunately, whole-genome sequencing of the mutant did not reveal any genetic polymorphism(s) suggestive of the target or MoA. Most of the analogues from the series were cross-resistant against this mutant except the thiadiazole analogue **3c**, indicating additional targets and/or a different MoA. Given the failure to identify genetic polymorphisms in the raised mutant, we considered an alternative target identification approach and accordingly decided on chemoproteomics, using *Mycobacterium bovis* BCG cell lysate. The compound **3d** was profiled on **3e**-immobilized beads. Subsequent affinity analyses suggested that the potential target could be BCG_3193 (Rv3169) with an apparent *K*_d_ value of 0.7 μM and a second weaker target BCG_3827 (Rv3768) with an apparent *K*_d_ value of 3.8 μM. The series was equally effective against the clinical strains of *Mtb* clinical isolates belonging to different lineages, exhibiting the MIC_90_ of 0.08–5 μM.

Next, a high-throughput screen of a Medicines for Malaria Venture (MMV) compound library comprising an ∼530 000 diverse set of compounds against *Mtb* was conducted at the National Institute of Allergy and Infectious Diseases of the National Institutes of Health (NIAID/NIH). The reconfirmed hits were profiled for MIC on multiple media conditions which identified a cluster of pyrazolylpyrimidinones represented by compounds **4** and **5** (Figures 5 and 6). Detailed SAR investigation resulted in improving the activity against *Mtb*, moderate to high aqueous solubility, and excellent in vitro microsomal stability. However, there was a narrow scope to improve the selectivity index against mammalian cell-line toxicity. Therefore, further optimization of pharmacokinetic properties along with improved mammalian cytotoxicity was needed to identify a compound suitable for in vivo efficacy studies. The compounds were bactericidal against replicating *Mtb* and retained good potency against clinical isolates, within 4-fold range of MICs against drug-sensitive *Mtb* strain. Transcription analyses of *Mtb* cultures treated with pyrazolylpyrimidinones revealed the upregulation of genes involved in iron-homeostasis. This was further verified by iron supplementation to the growth medium displaying increase in the MIC values, suggesting the perturbation of Fe-homeostasis as a MoA.^3^

In continuation of our TB drug discovery efforts, a whole-cell cross-screening against *Mtb* identified the 2-aminoquinazolinone series as an attractive chemotype to prosecute.^4^ Originally, the 2-aminoquinazolinones were synthesized for evaluation against the human malaria parasite *Plasmodium falciparum*, based on their structural similarity to a previously discovered antimalarial 2-aminopyridine series in our lab.^29^ The hit compound **6** exhibited good in vitro potency against *Mtb* but low solubility (MIC, 0.24 μM; solubility, <5 μM). The SAR investigation resulted in the identification of compound **7** with an improved solubility while maintaining potency (Figures 5 and 6). However, this series was not efficacious in a BALB/c mouse acute TB infection model despite the favorable pharmacokinetics profile. This intriguing discrepancy between in vitro vs in vivo efficacy was investigated thoroughly and found that this series was displaying a glycerol-medium dependent effect. Our MoA investigation efforts, involving SRM generation and whole-genome sequencing studies, validated this as point mutations mapped to glycerol metabolizing genes. This further reiterates the need for caution during screening, that one should consider the major difference in carbon metabolism between bacteria growing in standard TB culture medium containing glycerol compared to those found in TB-infected lungs.^26^

**Figure 7:**
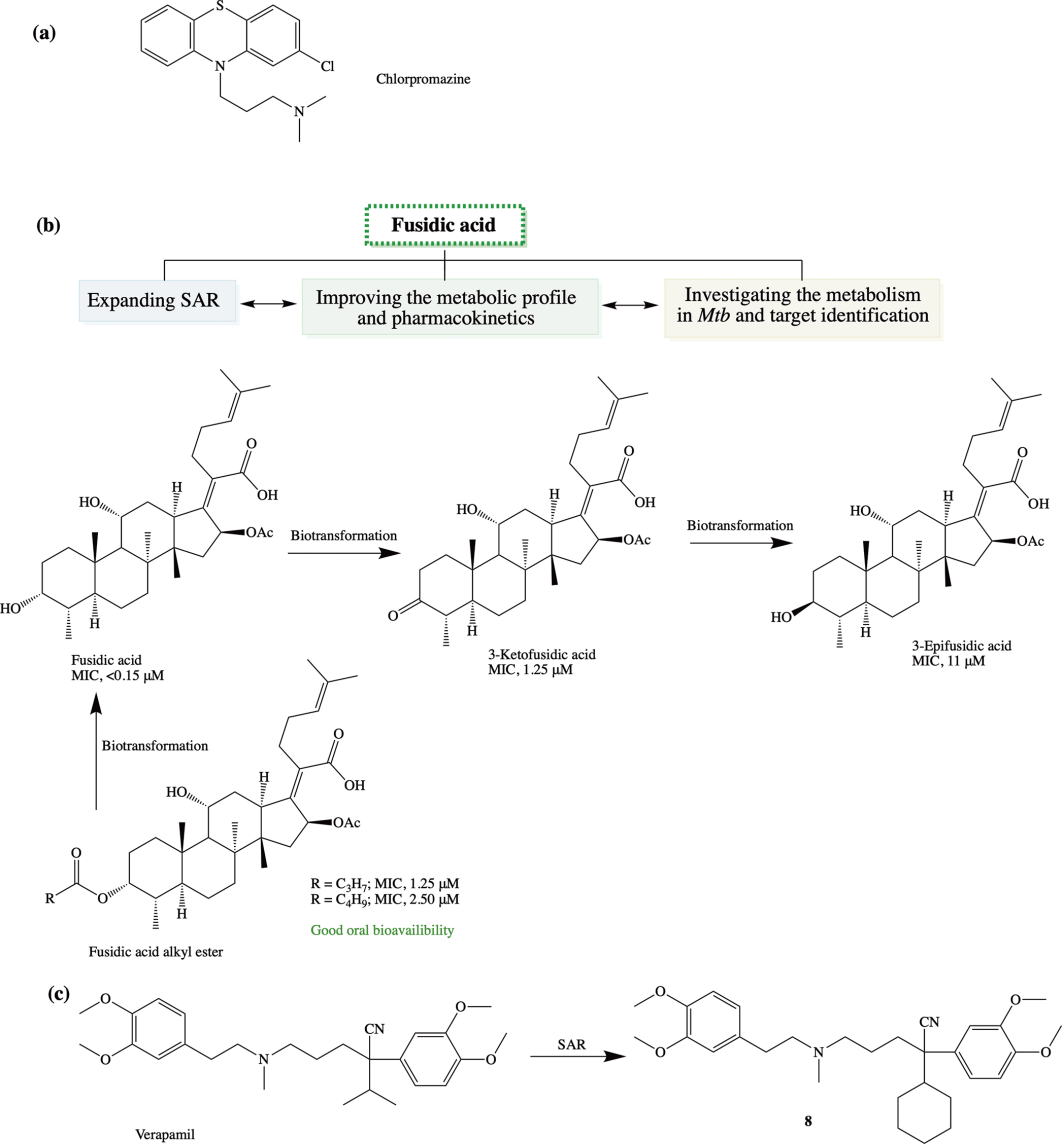
Addressing drug resistance using drug repositioning approaches: (a) chlorpromazine, (b) integrated approach investigating fusidic acid, and (c) verapamil.

Additionally, one of the strategies in tackling drug resistance is to reposition or repurpose existing drugs which can potentially reduce the cost and time of drug development.^30^ Toward this end, we first investigated the experimental drug chlorpromazine (CPZ) (Figure 7a), a phenothiazine originally developed for treatment of psychosis, and its metabolites against the mycobacteria in combination with a number of first- and second-line TB drugs and observed a potential for synergy with spectinomycin, kanamycin, STR, and 25-desacetylrifampicin (an active metabolite of rifampicin).^31^ Another drug we considered for repositioning is the triterpenoid antibiotic, fusidic acid (FA), due to its unique MoA, specifically, inhibition of bacterial protein synthesis through binding to elongation factor G in methicillin-resistant *Staphylococcus aureus*. It displayed good activity against both DS and DR clinical isolates of *Mtb*(32) and, therefore, was considered a viable candidate for repositioning for TB. We worked on the SAR of FA with respect to structural modifications that influenced in vitro potency, metabolic profile, and pharmacokinetics (Figure 7b).^33,34^ The rapid biotransformation of FA to its inactive epimer (3-epifusidic acid) via 3-keto fusidic acid in rodents complicated proof-of-concept studies in this model.^34^ A prodrug approach involving masking the metabolically labile C-3 position through esterification was found to improve absorption and tissue distribution of FA.^35^ To assess the molecular target of FA, our current efforts are focused on deconvoluting its MoA.

In recent times, use of efflux-pump inhibitors as an option to address drug resistance in TB has emerged. One such efflux-pump inhibitor is verapamil (VPL), a calcium channel blocker used clinically for the treatment of atrial fibrillation or atrial flutter, hypertension, cluster headache, and angina. VPL, as such, does not exhibit inhibitory activity against extracellular *Mtb* but enhances the anti-TB activity of other standard drugs, thus making it a potential candidate for treatment shortening and managing drug resistance. To this end, we investigated the SAR around VPL.^36,37^ Biological evaluation of VPL and norverapamil suggested that these compounds inhibit the expansion of *Mtb*-specific T cells. However, one promising analogue **8** inhibited intracellular *Mtb* replication without affecting the *Mtb*-specific T-cell expansion (Figure 7c). This analogue showed efflux-pump inhibition comparable to VPL in *Mtb*, and enhanced the inhibitory activities of isoniazid and rifampin on intracellular *Mtb*.^36^

Lastly, the most straightforward strategy in combating DR-TB is to identify novel drug pathway(s)/target(s) that could be exploited to effectively kill DR *Mtb* strains. In this context, we have identified and validated novel drug targets such as, GuaB2, encoding an essential inosine monophosphate dehydrogenase involved in guanine biosynthesis,^38^ WecA, an essential transferase of arabinogalactan biosynthesis,^39^ and Wag31, an essential protein in *Mtb* which has no known enzymatic activity but required for the proper assembly and function of the elongation machinery during cell division.^40^

## 6 Conclusion and Future Prospects

In the history of antibiotics, not a single drug yet discovered is evolution-proof. Sooner or later resistance will develop. Although *Mtb* exhibits a low mutation rate which is supported by its narrow genetic diversity, lack of a horizontal-gene transfer mechanism, and no added environmental benefit, the worsening epidemic of DR *Mtb* is a serious threat. This underscores the need to act now to stop the spread of these deadly DR strains. The resistance exhibited by *Mtb* to any TB drug is complex due to the contributory role of biological, clinical, and microbiological features: prescription vs nonadherence to the treatment, drug combination vs drug–drug interaction, granulomatous lesions vs suboptimal drug concentration, and intrinsic resistance vs acquired resistance. In addition to the traditional approach of discovering new drugs, other alternative approaches must be used in parallel. A significant effort in identifying key bacterial and host pathways and targets/proteins must be invested; this should cover investigations on persisters, exploring various networks associated with molecular mechanisms of persistence/tolerance. Similarly, a great deal of effort is needed in identifying new chemical entities using screening methods that closely mimic the host site environment. This is in view of the sobering reality around uncertainties in the biology of TB disease (and to a certain extent of its pathogen, *Mtb*), which complicates medicinal chemistry efforts toward optimizing a novel drug/regimen. Here, two alternative strategies could be (i) chemical optimization, allowing inactivated drugs to escape the resistance mechanisms, and (ii) targeting resistance, resistance mechanisms targeted by specific inhibitors that can resensitize resistant bacteria to the inactive drugs. We believe that the lack of information related to drug metabolism mediated by both host and *Mtb* enzymes significantly contributes to drug resistance via suboptimal dosing, poor patient compliance, subsequent treatment failure, and relapse. In this context, pharmacometabonomics, identifying drug-induced metabolome variations, can be useful. Another promising approach to potentially shorten the treatment duration and reduce the residual lung pathology/bacterial load is host-directed-therapy (HDT), which we have not covered in this Account. In addition, tailor-made regimens after accurate diagnosis should be practiced, and for this, cross-resistance, synergies, or antagonism among drugs of a range of possible combinations must be considered. Finally, more than ever, understanding the transmission of DR strains followed by a possible check is needed; this could be achieved by identifying desired genetic markers, supporting timely diagnosis of drug resistance in order to not present *Mtb* with the opportunity to evolve into a DR monster.
